# The Influence of Coal Tar Pitches on Thermal Behaviour of a High-Volatile Bituminous Polish Coal

**DOI:** 10.3390/ma15249027

**Published:** 2022-12-17

**Authors:** Valentina Zubkova, Andrzej Strojwas

**Affiliations:** Institute of Chemistry, Jan Kochanowski University in Kielce, 7 Uniwersytecka Str., 25-406 Kielce, Poland

**Keywords:** coal tar pitch, additives, co-carbonization, co-pyrolysis, interaction

## Abstract

The influence of three coal tar pitches (CTPs), having softening points at 86, 94, and 103 °C, on the thermal behaviour of a defrosted high-volatile coal during co-carbonization and co-pyrolysis was studied. The following research techniques were used: X-raying of the coked charge, TG/FT-IR, ATR and UV spectroscopies, extraction, SEM, STEM, and XRD. It was determined that CTP additives change the structure of the coal plastic layer, the thickness of its zones, and the ordering degree of the structure of semi-cokes to a different extent and independently from their softening points. The softening points of CTPs do not influence the composition and yield of volatile products emitted from blends with pitch as well as the composition, structural-chemical parameters, and topological structure of material extracted from coal blends. It is suggested that such a lack of existence of any correlation between the softening points of CTPs and the degree of their influence on the thermal behaviour of coal was caused by the presence of the atoms of metals (Fe and Zn) in the CTPs. These atoms change the course of the carbonization of the CTPs themselves and their influence on organic substance of coal in blends with CTPs.

## 1. Introduction

The changes in the situation on the global coal market and the diversification of importers of coking coals set a task for the national industry to ensure the work continuity of Polish coking plants. The research conducted by Miroshnichenko et al. [1], in different seasons, suggests that in winter the same coal blends improve their thermoplastic and coke-making properties. Our previous research, conducted on a frozen high-volatile coal, showed that after defrosting this coal significantly improves its thermoplastic properties and, during carbonization, forms the gas-saturated zone in the plastic layer [2]. However, compared to a fresh one, a defrosted coal emits more volatile products during heating. This fact lowers the profitability of the coke-making process of such coal. 

The research conducted by Nomura and Arima [3] proved that the addition of a coal-derived binder can reduce the yield of volatile products from the blend during pyrolysis at the temperature below 450 °C. Coal tar and coal tar pitch (CTP) have been known for a long time as additives that improve the fluidity of coals [4,5,6,7,8] and their caking properties [3,4]. The authors of the works [9,10] suggest that pitch additives can be used as additives that bring back the lost thermoplastic abilities to low-oxidized coals. These additives can be used as binders for briquettes with coals having worse caking properties [11,12,13,14]. CTPs extend the plasticity range and lower the softening temperature of coals [15,16]. 

Despite a large amount of research on the influence of CTPs on the course of the coking process, Nomura [17] suggests that the mechanism of their influence on coal substance has not been fully explained so far. According to the authors of the works [18,19], CTPs in blends with coals participate in the processes of “hydrogen shuttling” being either hydrogen donors or acceptors. It is believed that the decomposition products of CTP interact with coal and increase its thermoplasticity this way [3,20]. As a result of such interaction, an increase in the coking pressure of coal blends with pitch can take place [10,21,22,23]. Duffy et al. [15] determined an increase in the coking pressure as a result of the addition of pitch to a coal of higher fluidity. However, in Nomura’s opinion [17], CTP additives can reduce coking pressure. Previous research [16] showed that the addition of CTP to coals, having a distinct content of volatiles, changes the composition of the material extracted from them in different way. In this research, attention was attracted to the fact that the addition of CTP to coals as a material containing polycyclic aromatic hydrocarbons (PAHs) led to a decrease in content of these PAHs in the extracts of plasticized coal blends.

The aforementioned shows that, despite numerous investigations connected with the use of CTPs as additives to coked coals, the phenomenon of their interaction with coal substances has not been explained fully. In order to extend the knowledge about the interaction of CTPs with coals and to specify the effect of their influence on the mechanism of changes taking place in coked coal, the research had to be conducted with the use of three CTPs having the softening points distinct but close. It was expected that the continuity of specific changes in the thermal behaviour of coal would correspond to the changes in the softening points of CTPs. Due to this, the impact effect of CTPs and their softening points on thermochemical changes in organic substance of coal would be easily determined. 

The specific aims of the research were to study the influence of CTPs on:

− the changes in thickness of plastic layer and its zones and the changes in structure and texture of the material of carbonized charge;
− the changes in volume of heated charge;
− the course of coal pyrolysis and the composition of volatile products of pyrolysis;
− the yield of material extracted from the zones of plastic layer and structural chemical parameters of this material.

## 2. Materials and Methods

### 2.1. Characteristics of Studied Samples

The subjects of the investigation were blends of a commercial sample of defrosted high-volatile bituminous coal with coal pitches. The characteristics of studied coal are presented in Table 1.

A fresh sample of commercial coal was frozen and stored at a temperature of −15 °C for 15 months. The coal was defrosted at room temperature. After drying to the air-dry state, the coal was ground to the grain size of <3 mm. The commercial samples of hard CTPs–CTP86BL (CTP86 in the text) and CTP103BL (CTP103 in the text) along with experimental pitch CTP94 were used for the investigation. The characteristics of CTPs are presented in Table 2.

The softening point of coal tar pitch was determined according to the ISO 5940-2:2007 standard using the Standard Test Method for Softening Point of Pitches (Mettler Softening Point Method, ASTM D3104).

The CTP samples were ground to the grain size of <0.2 mm in diameter. The samples of 2 wt.% of CTP were prepared from the samples of coal and CTPs using the standard procedure of the manual mixing of powder ingredients previously described in [16]. 

### 2.2. Carbonization Test

**Figure 1:**
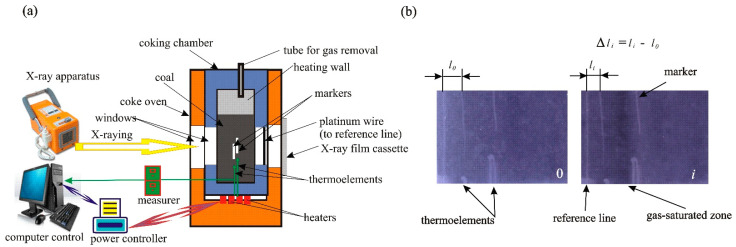
A scheme of the laboratory unit to study the carbonization process with X-raying (**a**) with X-ray pictures of heated charge of coals (**b**).

The carbonization of coal and its blends with CTPs was carried out in a laboratory unit in order to study the coking process using X-raying according to the methodology described previously [24]. Figure 1 presents a scheme of the laboratory unit used to study the course of the coking process with X-raying.

The unit consists of an oven with one-sided vertical heating and a coking chamber. There are some windows in the side walls of the chamber and oven through which the coked charge is X-rayed. For this purpose, an ORANGE 1040 HF X-ray apparatus was used that worked in the mode of U = 85 kV, 25 mAs. The mass of coal charge was 480 g., and the sizes of the coal grains were below 3 mm. The changes in temperature of the charge and the course of the coking process were controlled by a computer (Figure 1a). The markers, the positions of which were registered in the X-ray images taken during carbonization, were placed in the carbonized charge. The changes in position of Δ*l* markers were calculated on the basis of elaboration of these images by a CorelDRAW version X4 software. The values of changes in position of markers were calculated by the formula: Δ*l = l_o_ − l_i_.* The measurements were made with respect to the vertical reference line (Figure 1b) at the designated height of charge. The marker was placed in the charge in the distance of 36 mm from the heating wall of the coking chamber. The changes in the position of the markers, depending on the temperature, reflected the changes in the volume of the charge layer between the heating wall and the markers at different temperatures of carbonization. The carbonization was carried out to reach a temperature of 950 °C on heaters with the heating rate of 4 °C min^−1^. After this temperature was reached, the coking chamber with charge was cooled rapidly. After cooling, the charge was prepared in order to separate the zones in the composition of the plastic layer of coal. The thickness of this layer and the thickness of its zones were measured in the X-ray images taken during the penetration of the charge by a needle thermoelement according to the methodology described previously [24].

### 2.3. Pyrolysis in a TG/FT-IR Unit

The samples of the defrosted coal, CTPs, and blends of coal with CTPs, with a grain size of <0.2 mm, were pyrolyzed under the nitrogen atmosphere of high purity to the temperature of 750 °C according to the methodology described in the work [25]. The samples, having approximately 25 mg in weight, were heated in a platinum crucible of a Q50 thermobalance with the heating rate of 4 °C⋅min^−1^. The formed volatile products were directed to the interface via a transfer line and next to a Nicolet iS10 spectrometer Madison, WI, USA). This way, the FT-IR spectra of the volatile products were registered in the wavenumber range of 4000–600 cm^−1^. The elaboration of the FT-IR spectra was made by an OMNIC 9 software. 

### 2.4. Extraction of Plasticized Samples

The extraction of plasticized samples of coal and its blends with CTPs was conducted using a mixture of chloroform and methanol (50:50) in an ultrasonic bath for 10 h at room temperature. After the extraction was completed, the extractant was distilled under a vacuum. The residue after distillation was kept in a vacuum dryer at a temperature of 25 °C until the constant weight was reached. The yield of extraction was calculated on the basis of mass of the obtained extract. The swollen grains, maximally swollen grains, and the material from the gas-saturated and compacted zones were treated according to the same procedure [25]. The material condensed on the surface of the swollen grains of the carbonized charge [26] was rinsed from that surface. The material extracted from the gas-saturated and compacted zones of the plastic layer was also obtained.

### 2.5. Spectroscopic Investigation 

The samples of material that are soluble in the chloroform-methanol mixture were studied by a Nicolet iS10 spectrometer using a Smart MIRacle module (the ATR technique). In this investigation, a diamond monocrystal was used. The spectra were registered in 64 scans in the wavenumber range of 4000–600 cm^−1^. The registered spectra were normalized with regard to the band near 1640 cm^−1^, which was present in the spectra of all samples. The ATR spectra were elaborated by an OMNIC 9 software. The baseline was corrected in order to eliminate the non-specific background. 

The same samples of extracted material, weighing 0.0001 g, were dissolved in 50 mL of acetonitrile. The UV spectra were obtained using a JASCO V630 spectrometer (Tokyo, Japan) and normalized at the wavelength of 195 nm, and next, the deconvolution option was applied using a Spectra Manager software (version 2.08.04).

### 2.6. Microscopic Investigation of Obtained Samples

The samples separated from the gas-saturated zone and the zone of maximally swollen grains of coal were studied using a Quanta 3D FEG scanning electron microscope (SEM, Boynton Beach, FL, USA) with an accelerating voltage of 5 keV. The samples of the material extracted from the gas-saturated zone were studied with the use of a FEI Tecnai Osiris transmission electron microscope (Lincoln, NE, USA) with an X-FEG Schottky field emitter. The accelerating voltage was 200 kV [25].

### 2.7. X-ray Diffraction Investigation 

The X-ray phase analysis of solid residues was carried out using an internal standard. As a standard, a powdered NaF (ACS reagent, ≥99% produced by Sigma-Aldrich, St. Louis, MO, USA) was applied, the (002) line of which is near the (002) line of the studied residues by its angular position. A pyrolytic graphite powder GPR^TM^ (produced by BDH Laboratory Supplies,) Poole, UK, having a turbostratic lamellar structure, was a model substance.

The semi-coke samples from coal and its blends with CTPs heated to a temperature of 650 °C were mixed with 10% of NaF. After the obtained blends were ground in an agate mortar, some rods, of 0.58 mm in diameter and approximately 13 mm in height, were formed from them. The samples prepared this way were studied using a polycrystal diffractometer in the mode U = 25 kV and I = 40 mA. The period of counting impulses was 10 s. The diffractograms were registered in the range of angles of 2Θ 15–42. The interplanar distances *d*_002_ and the amount of crystalline phase *C_cryst_* were calculated according to the methodology presented in work [26].

The *C_cryst_* parameter was calculated according to the formula:

$$C_{cryst}=\frac{P\cdot 100}{k\cdot x\cdot \left(100-P\right)}$$

where *P* is the amount of internal standard (NaF) in the test sample, *x* is the ratio of the integral intensity of the (002) line of the internal standard (NaF) to that of the test sample, and *k* is an experimentally obtained coefficient from the calibration curve. The experimentally determined coefficient *k* is equal to 0.88.

The interplanar distances (*d*_002_) were determined according to Wulff–Bragg formula:

$$d_{002}=\frac{n\cdot \lambda }{2\cdot sin\;\theta }$$

where *n* is the diffraction order (*n* = 1), λ is the wavelength of radiation (*λ* = 1.5406 nm), and *θ* is the angle of scattering.

## 3. Results and Discussion

### 3.1. Influence of CTP Additives Having Different Softening Points on the Course of Co-Carbonization Process of Defrosted Coal

**Figure 2:**
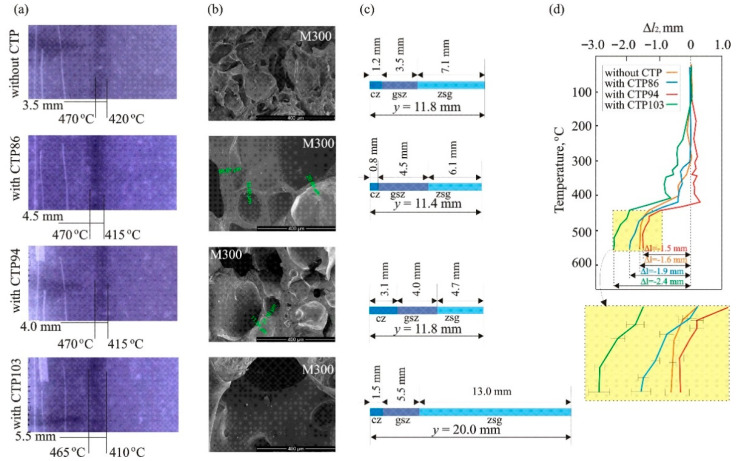
Results obtained during carbonization of coal and its blends with CTPs: (**a**) X-ray films of carbonized charge; (**b**) SEM images of gas-saturated zones in carbonized charge; (**c**) composition of plastic layers in carbonized charge; (**d**) changes in position of the marker in the charge of carbonized coal and its blends with CTPs.

Figure 2 presents the results obtained during the carbonization of coal and its blends with CTPs. There is a dark band visible in the X-ray images that is a projection of the gas-saturated zone on the X-ray film (Figure 2a). The X-ray images imply that the addition of every CTP to defrosted coal causes an increase in the thickness of the gas-saturated zone in the plastic layer of blends. The greatest thickness of these zones was observed for the blend of coal with CTP103. The SEM images of material from this zone (Figure 2b) prove that the zone is filled with some foam-like plastic mass. The SEM images (taken at a magnification of M300) imply that the cells of the plastic mass in the charges of coal and its blends with CTPs have different dimensions. The thickness of the cell walls also differs. The cells in the blend with CTP 103 have the largest sizes. The thickness of the plastic layer of coal (y) and its blends with CTPs is also different (Figure 2c). The thickness of the zones of the plastic layer (swollen grains, gas-saturated, and compacted ones) also differs. The addition of CTP86 causes a decrease in the thickness of the plastic layer but the addition of CTP103 causes an increase. Figure 2d presents the changes in position of the marker in the charge of carbonized coal and its blends with CTPs. 

These changes point out to changes in the volume of the layer of the heated charge between the marker and heating wall. The shape of the curve Δ*l = f(T)* in Figure 2d does not indicate any existence of a correlation between the changes in volume and softening points of CTPs. A greater shift in position of the marker, towards the negative values of Δ*l* at the temperature of 550 °C, is observed in case of blends with commercial pitches, mainly with CTP86. This implies a greater volume reduction of the heated charge at the stage of its re-solidification and, hence, a greater density and compactness of the obtained semi-coke. The addition of CTP94 does not influence changes in the position of the marker towards the negative values of Δ*l* at a temperature of 550 °C in contrast to coal without additives (Figure 2d).

### 3.2. Influence of CTP Additives Having Different Softening Points on the Course of Their Co-Pyrolysis with Defrosted Coal

**Figure 3:**
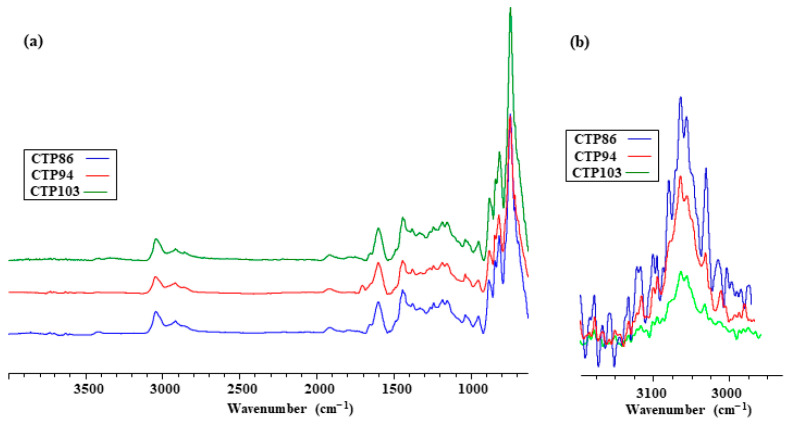
ATR spectra of CTPs (**a**) and fragments of FT-IR spectra of their volatile products of pyrolysis emitted at a temperature of 300 °C (**b**).

Figure 3a presents the ATR spectra of the studied CTPs and the fragments of the normalized FT-IR spectra of their volatile products of pyrolysis emitted at a temperature of 300 °C. 

Analyzing the composition of CTPs, Diez et al. [27] stated that there are approximately 10,000 various chemical compounds having a distinct molecular mass present in them. It follows from the ATR spectra in Figure 3a that the presence of identical functional groups and groups of atoms is characteristic of the CTPs used in this research. Therefore, it was expected that these CTPs would interact with the organic substance of coal in the same way. Taking into account the differences in softening points of CTPs, it was assumed that the compounds of greater molecular mass present in CTPs having higher softening points would be included in the composition of volatiles at higher temperatures of pyrolysis. This assumption is confirmed by a fragment of the FT-IR spectra of volatile products emitted at a temperature of approximately 300 °C: more hydrocarbons are emitted from CTP86 than from CTPs having higher softening points. This implies that, under the influence of products emitted from CTP86, coal grains would soften at lower temperatures. This softening should cause an increase in the thickness of the zone of swollen grains in the plastic layer of the blend of coal with CTP86. However, it did not. It follows from Figure 2c that the zone of swollen grains in the plastic layer of the blend with CTP103 has the greatest thickness.

**Figure 4:**
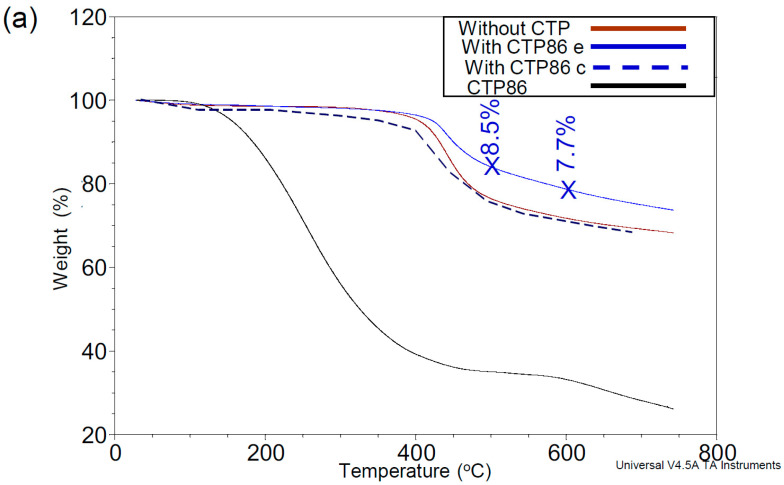

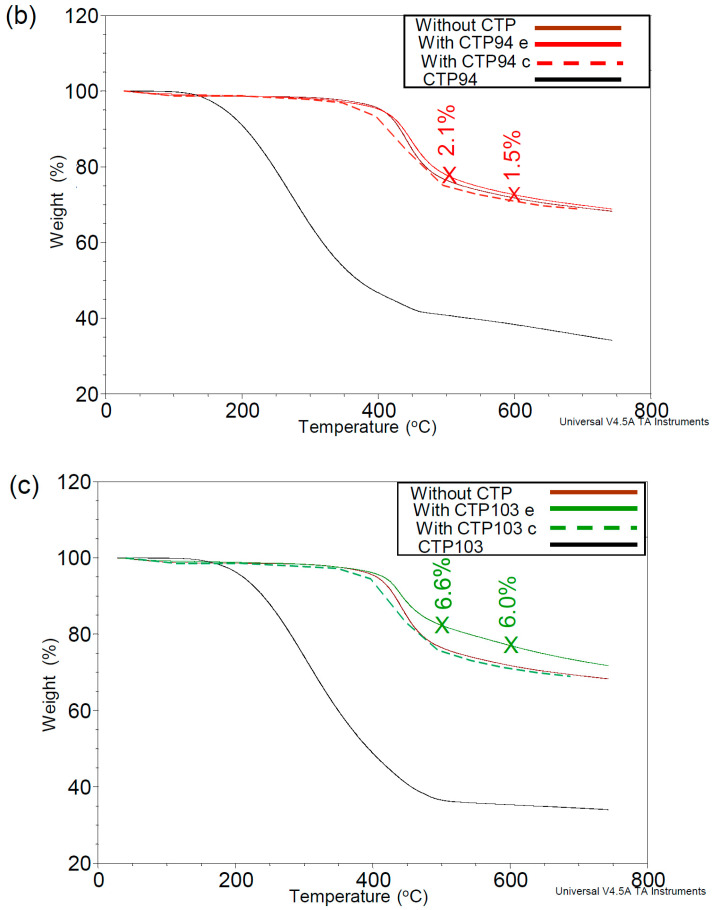
TGA curves of coal, its blends with CTPs and the CTPs: CTP86 (**a**), CTP94 (**b**), and CTP103 (**c**).

According to the conclusions made by the authors of works [3,20], the decomposition products of CTPs should interact with the surface of coal grains that have not yet softened. In order to prove these statements, Figure 4 presents the TGA curves of coal, CTPs, and their blends. In this figure, the course of TGA curves of blends clearly suggests that CTPs interact with coal substance in different way. This figure also gives the mass loss curves of coal blends with CTPs calculated at different temperatures based on the additivity rule according to the formula:

$$CBW\left(\%\right)=CW\left(\%\right)\cdot 0.98+CTPsW\left(\%\right)\cdot 0.02$$

where *CBW* (%) is the calculated weight of blend of coal with CTPs, *CW* (%) is the experimentally measured weight of coal, *CTPsW* (%) is the experimentally measured weight of coal tar pitch, and 0.98 and 0.02 are the relative proportions of coal and CTPs in the mixtures. The results of the calculations are shown in Table S1 in the Supplementary Materials.

The values of the difference in the mass loss of experimental curves and those calculated taking into account the additivity rule are presented in the TGA curves. The values of deviations from the additivity rule were given for the temperatures of 500 and 600 °C. The experimental decrease of the mass loss for the blend of coal with CTP86 at these temperatures is 8.5 and 7.7% appropriately, for the blend of coal with CTP94–2.1 and 1.5%, and for the blend of coal with CTP103–6.6 and 6.0%. 

It follows from the shape of the curves in Figure 4a,c that at temperatures above 400 °C, the CTP86 and CTP103 additives cause a decrease in the mass loss of blends compared to the mass loss of coal without additives. However, the experimental curve of blend with CTP94 almost coincides with the TGA curve of coal without additives (Figure 4b). CTP86 additive has a greater influence on the decrease in the mass loss of the blend.

**Figure 5:**
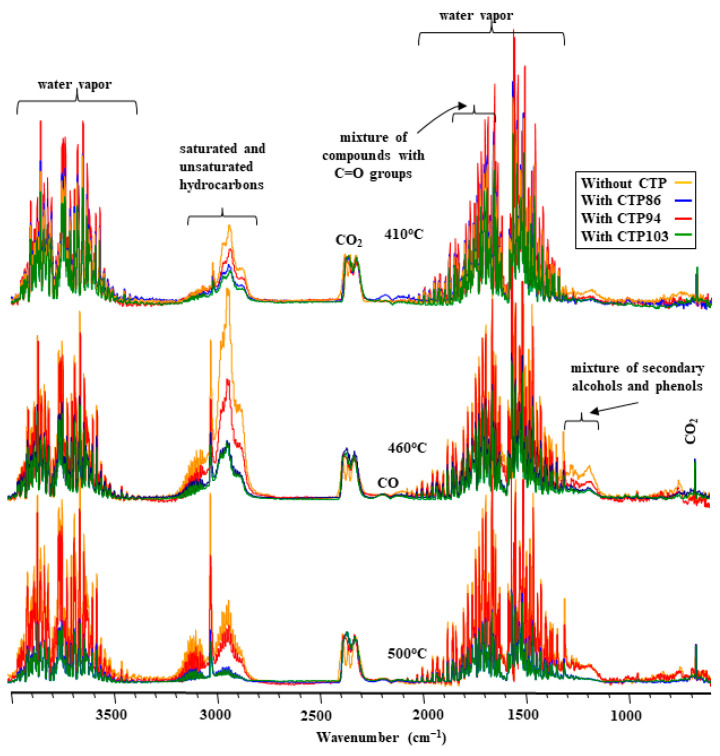
FT-IR spectra of volatile products emitted from coal and its blends with CTPs at pyrolysis temperatures of 410 °C, 460 °C, and 500 °C.

Figure 5 presents the normalized FT-IR spectra of volatile products emitted from the blends of coal with CTPs at pyrolysis temperatures of 410 °C, 460 °C, and 500 °C. 

There are some differences in the composition of volatile products emitted from the blends at a temperature of 410 °C visible in these FT-IR spectra. This temperature corresponds to the occurrence of the zone of maximally swollen grains in the plastic layers of blends. In the FT-IR spectra of the volatile products from the blends in the wavenumber range of 3200–2800 cm^−1^, the heights of the bands of saturated and unsaturated hydrocarbons are different. However, the heights of these bands do not show any dependence from softening points of CTPs. The greatest contribution ratio of hydrocarbons is observed in the composition of the volatiles of defrosted coal. Among the volatile products from blends of coal with CTPs, the blend of coal with CTP94 has a greater contribution ratio of hydrocarbons. 

Nomura and Arima [3] stressed that the products of decomposition of coal-derived binder cause the plasticization of coal grains. Koch et al. [28] also suggested that volatile products of decomposition can migrate to the cold side of the oven and impregnate coal grains. The plasticizing influence of CTPs and the impregnation of grains should have found their reflection in the changes of relief of plasticized grains. Taking the aforementioned into account, it was reasonable to analyse the effects of this interaction with the coal grains in the zone of swollen grains of plastic layer and to present the characteristics of the material condensed on grains. 

**Figure 6:**
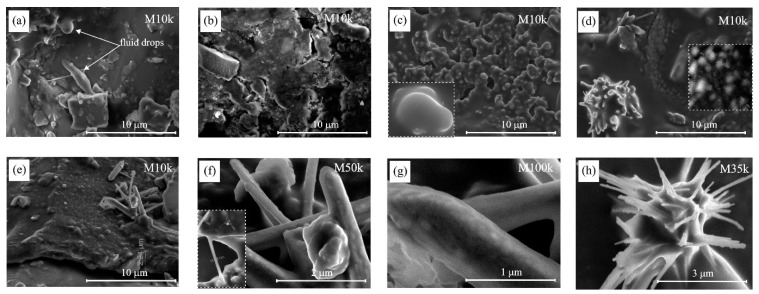
The SEM images of fragments of surface of swollen grains from the charge of coal and its blends with pitches. (**a**) surface of swollen grain in the charge of coal without CTPs, (**b**) surface of swollen grain in the charge of coal blend with CTP86, (**c**) surface of swollen grain in the charge of coal blend with CTP94, (**d**) surface of swollen grain in the charge of coal blend with CTP103, (**e**) crust on the surface of swollen grain in the blend with CTP103, (**f**–**h**) nano-objects on the surface of swollen grains in the charge of coal blend with CTP103.

Figure 6 presents the SEM images of the fragments of the surface of swollen grains from the charge of coal and its blends with pitches. In contrast to the swollen grains of defrosted coal, there wasn’t any presence of fluid drops detected on the surface of grains from the charge of blends with CTPs [2], i.e., there were no traces of leakage of thermobitumen from the swollen grains found in the charges of blends with CTPs. 

It follows from Figure 6b,d that CTP additives, having distinct softening points, change the relief of the surface of swollen grains in different ways. This indicates that the volatile products formed during the carbonization of coal and blends interact with the surface of coal-swollen grains differently. This interaction results in the appearance of ‘erosion’ areas on the surface of the swollen grains in the charges of blends with CTP86 and CTP94 (Figure 6b,c) and the formation of a crust with nano-objects on the surface of swollen grains in the blend with CTP103 (Figure 6d–h). On the surface crust of the swollen grains from the blend with CTP 103 (Figure 6e), there are some nano-objects visible in the form of a bunch of tubes. The surface of grains from the blend with CTP94 (Figure 6c) and the tubes in Figure 6e look like clusters of connected globules approximately 100–200 nm in size (Figure 6g).

In the FT-IR spectra at a temperature of 460 °C (Figure 5), which corresponds to the occurrence of the gas-saturated zone in the plastic layer, the differences in height between the bands of hydrocarbons in the wavenumber range of 3200–2800 cm^−1^ and the bands in the range of 1300–1100 cm^−1^ become more explicit. A greater contribution ratio of saturated and unsaturated hydrocarbons is characteristic of volatile products of pyrolysis of coal without CTP additives. Wide bands that point out to the presence of hydrocarbons in the composition of volatiles from the blends of coal with CTP86 and CTP103 coincide. However, the bands of hydrocarbons, which originate from volatile products of the blend with CTP94, take the middle position. The FT-IR spectra of the volatile products imply that there is no dependence between the shape of the spectra and the softening points of the CTPs observed. 

**Figure 7:**
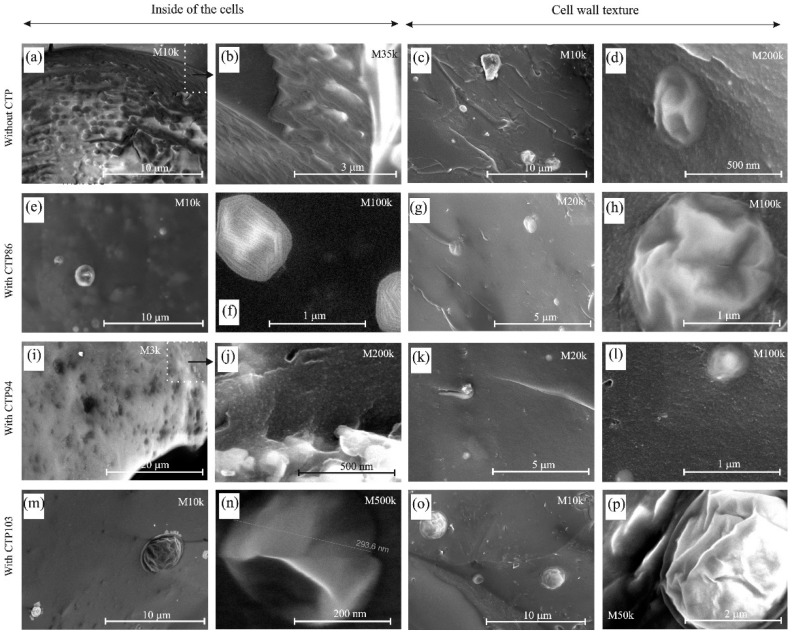
The SEM images of the material from the gas-saturated zone of coal and its blends with pitches. (**a**,**b**) insides of cells in gas-saturated zone of coal without CTPs, (**c**,**d**) cell wall textures in gas-saturated zone of coal without CTPs, (**e**) inside of cell in gas-saturated zone of coal with CTP86, (**f**) inside of cell in gas-saturated zone of coal with CTP86 with nano-objects, (**g**,**h**) cell wall textures in gas-saturated zone of coal with CTP86, (**i**,**j**) insides of cell in gas-saturated zone of coal with CTP94, (**k**,**l**) cell wall textures in gas-saturated zone of coal with CTP94, (**m**) inside of cell in gas-saturated zone of coal with CTP103, (**n**) inside of cell in gas-saturated zone of coal with CTP86 with nano-objects, (**o**,**p**) cell wall textures in gas-saturated zone of coal with CTP103.

Volatile products that are formed and cumulated in the cells of the plastic mass can interact with each other and the material of the cell walls. Figure 7 presents the SEM images of the interior of cells of the material from the gas-saturated zone.

At a magnification of M10k, on the surface of the interior of a cell of the plastic mass of coal without CTP (Figure 7a), there are some round concavities that were not found inside the cells of blends with CTPs. The interior of a cell of the gas-saturated zone from the blend with CTP 94 shows the signs of the gasification of the material, even at a magnification of M3k (Figure 7i). The interior of the cells in the gas-saturated zone from blends with commercial CTPs looks more compact but there are some flat nano-objects resembling yarn hanks present on the surface of a cell from the blend with CTP86. This may be the pleated balloons, the folds of which are regularly arranged. The change in direction of the arrangement of folds takes place at an angle of 120 degrees (Figure 7f). On the surface of a cell of plastic mass in the blend with CTP103, there also were some balloons that were partly deformed and partly round and empty inside (Figure 7n).

The texture of the material of the cell walls of the plastic mass of coal with CTPs (Figure 7g,k,o) has a more compact appearance than that without CTP additives. It is characteristic that in the material of the cell walls, there are some objects that resemble deformed balloons of various shapes and sizes. The fact that the balloons remained non-crushed on the surface of a broken sample points to a greater mechanical resistance of their material. It is suggested that the films making balloons were formed as a result of the condensation of the hydrocarbons from volatile products created in the gas-saturated zone of coal and its blends with CTPs. The formation of these films, as a result of polymerization of compounds present in the composition of volatile products, could have been one of the reasons for a decrease in contribution of saturated and unsaturated hydrocarbons in the composition of volatile products of pyrolysis of coal with CTPs. The permanent formation and the closing of cells in the plastic mass of coal and its blends with CTPs could have caused the presence of balloons in the cell walls of the plastic mass.

At the temperatures of occurrence of the compacted zone (T = 500 °C), the bands originating from the blends of coal with CTP86 and CTP103 coincide with the FT-IR spectra of volatiles (Figure 5), as well as the bands originating from the defrosted coal and its blend with CTP94 which almost overlap. The shape and height of the bands of normalized FT-IR spectra of the blends of coal with CTPs in the range of 3100–2800 cm^−1^ (Figure 5) do not show any continuity of changes that would imply the dependence of these changes from the softening point of CTPs. 

### 3.3. Analysis of Condensates and Extracts

Table 3 presents the yields of material that was obtained during the treatment of plasticized samples by ultrasounds in the chloroform-methanol mixture. 

The data presented in Table 3 imply that the amounts of the material rinsed from the surface of grains and extracted from the gas-saturated and compacted zones are distinct but do not depend on the softening point of CTPs. The addition of commercial pitches (CTP86 and CTP103) causes a decrease in the amount of obtained material compared to the yield of material from defrosted coal. The only exception is the material from the compacted zone. In contrast, the addition of CTP94 causes an increase in the amount of the obtained material.

Shui et al. [29] pointed out to the existence of a dependence between the amount of extracted material and the degree of coal plasticization. It follows from Figure 2a,c that the thickness of the gas-saturated zone in the plastic layer of coal with CTPs increases, i.e., the range of appearance of the viscous-liquid state in plastic layer widens. On the other hand, the yield of the material soluble in the chloroform-methanol mixture in the presence of CTP86 and CTP103 additives decreases. It was suggested in previous research [2] that the defrosting of coal causes the mechanical destruction of the organic substance of coal and the formation of radicals. The formed radicals can easily interact with hydrocarbons with low ionization potential in the volatile products of decomposition of CTPs and initiate the reactions of dimerization and polymerization taking place with their participation [30]. In this way, compounds of greater molecular mass are formed. These compounds are not extracted by the chloroform-methanol mixture but, despite this, they play the role of plasticisers of the organic substance of coal. A similar phenomenon of the influence of pitch on a decrease in the amount of polycyclic aromatic hydrocarbons in the material extracted from plasticized coals was stated in previous research [16]. The values of the yield of the material soluble in the chloroform-methanol mixture in Table 3 cannot be considered as dependent on the softening point of CTPs.

**Figure 8:**
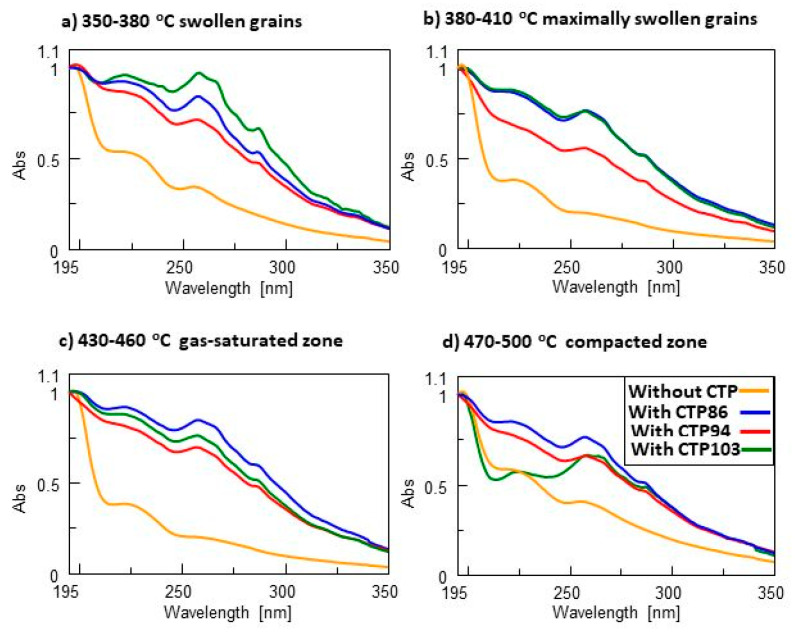
The normalized UV spectra of material that was rinsed from the surface of swollen grains (**a**,**b**) and extracted from the gas-saturated and compacted zones (**c**,**d**).

Figure 8 presents the normalized UV spectra of material that was rinsed from the surface of swollen grains (Figure 8a,b) and extracted from the gas-saturated and compacted zones (Figure 8c,d). In the spectra of the material from the charge with CTPs that was rinsed from the layer of grains heated to the temperature of 350–380 °C (Figure 8a), there are same types of compounds with chromophore groups and unsaturated bonds that are present in the material of defrosted coal without additives. However, the shape of the normalized spectra implies that the addition of CTP103 causes the appearance of a higher absorbance in the UV spectra (the range of 225–325 nm). This points to a greater concentration of compounds with chromophore groups. 

The material from the zone of maximally swollen grains from the charge with CTP86 and CTP103 additives (Figure 8b) has the same absorbance in the UV spectra. Among the materials from the gas-saturated and compacted zones, the material with the CTP86 additive (Figure 8c,d) has the highest absorbance in the UV spectra. In terms of absorbance, the material with the CTP94 additive takes the lowest position. However, compared with the UV spectrum of material from the charge without additives, its position is higher. This tendency does not repeat itself for the material from the compacted zone because the material from the blend with CTP 103 additive has a lower absorbance in the range of wavelength of 195–225 nm. It implies the absence of cyclic compounds and aromatic rings with chromophore groups in this material. 

**Figure 9:**
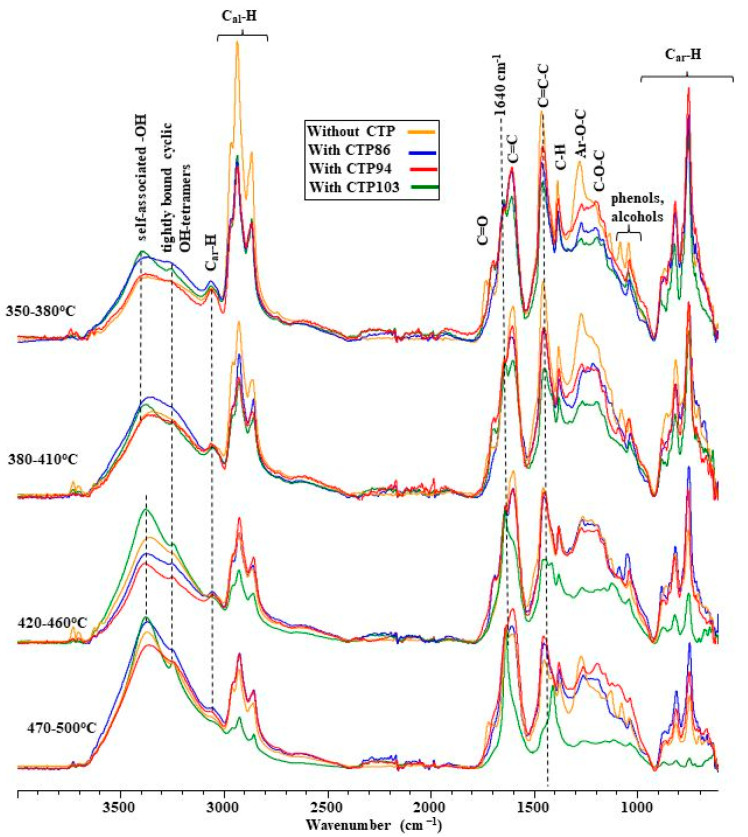
ATR spectra of material that was rinsed from the surface of grains and extracted from the gas-saturated and compacted zones.

Figure 9 presents the ATR spectra of material that was rinsed from the surface of the grains and extracted from the gas-saturated and compacted zones. The comparison of shape of the normalized ATR spectra shows that the contribution ratio of the groups of C_al_-H type in the range of 3000–2800 cm^−1^ decreases in the material condensed on the surface of grains from the charge with CTP additives. In the wavenumber range of 3600–3000 cm^−1^, the contribution of H-bonds (namely self-associated −OH) increases in the material from blends with commercial pitches. In the material extracted from the blend with CTP103, the contribution ratio of bonds of C_al_-H type (the range of 3000–2800 cm^−1^) decreases and the contribution ratio of H-bonds (self-associated -OH and tightly bound cyclic OH-tetramers) increases [31]. 

In the ATR spectra of the material condensed in the wavenumber range of 1800–1600 cm^−1^, there are some bands that point out to the presence of saturated and unsaturated esters (the exception is the material from the charge of coal with CTP86). In the material extracted from the blend with CTP103, the bands attesting to the presence of aromatic rings (C=C bonds near 1600 cm^−1^) and the bands indicating the presence of esters disappear in this range. In the material from the blend with CTP103 in the fingerprint range of 1400–1000 cm^−1^, the absorbance of all bands decreases gradually with the rise of temperature and the bands corresponding to the presence of deformation stretches of C_ar_-H type in the range of 900–600 cm^−1^ disappear. The disappearance of bands of these stretches in the extract from the gas-saturated zone of blend with CTP103 implies that dehydrocracking processes of substituted aromatic compounds were activated. Hydrogen released during these processes can participate in the reactions of its disproportionation. The shape of ATR spectra that are presented in Figure 9 does not point to the appearance of any continuity of the changes in structural-chemical parameters for studied samples with regard to the softening points of CTP additives.

**Figure 10:**
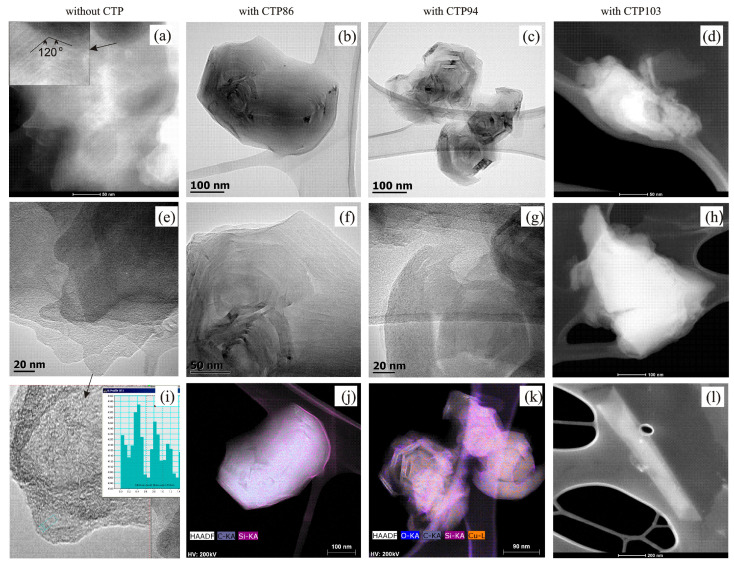
STEM-HAADF images of material that was extracted from the gas-saturated zones of investigated charges of coal and its blends with CTPs.

The utmost differences in shape of the bands corresponding to the appearance of hydrogen bonds are observed in the ATR spectra for the extracts from the gas-saturated zone. This implies that there are some differences in the ability of the material of the extract to aggregate and form supramolecular structures. Figure 10 presents the results of the research on material that was extracted from the gas-saturated zones of investigated charges of coal and its blends with CTPs with the use of a scanning transmission electron microscope (STEM). 

The visualization of the extracted material implies that the compounds present in it are able to form various topological structures (Figure 10a–l). It follows from the STEM-HAADF images that the material extracted from the gas-saturated zone of coal without CTP additives consists of several superimposed layers. In these layers, structural elements are arranged parallel to each other but at an angle of approximately 120° with respect to the elements of neighbouring layers (Figure 10a). Such arrangement of the elements resembles topological structures similar to the smectic mesophase. 

It was determined on the basis of the measurements made in the TEM mode (Figure 10e) that the distance between the chains put together on the edges of the layers is 0.39 nm (Figure 10i). In the material extracted from the zones of the plastic layer of coal with CTP86 (Figure 10b,f) and CTP94 pitches (Figure 10c,g), there were some layers twisted similar to supramolecular structures of axialites [32]. The images in Figure 10b,f and Figure 10c,g show that the angle of twisting of layers is near to 120 degrees. The particles of the material extracted from the gas-saturated zone from the blend of coal with CTP94 have sizes smaller than the particles in the extract from the blend with CTP86. The elemental mapping proves that axialites from the blend of coal with CTP86 contain C and Si atoms (Figure 10j), whereas axialites from the blend of coal with CTP 94 (Figure 10k) contain C, Si, O, and Cu atoms. There were no topological structures and layer arrangement found in the material extracted from the blend of coal with CTP103. It is known that the interaction of polymer with low molecular weight compounds having mesophase properties leads to the occurrence of mesophase phenomenon in the polymer itself [33]. The nature of low molecular weight material present in the gas-saturated zone (the capability of formation of supramolecular structures) may give the plastic mass of the blends with CTP86 and CTP94 some unusual properties that determine the thermal behaviour of coal during the carbonization process. The material extracted from the gas-saturated zone of the blend with CTP103 does not show such properties. The disappearance of bands in the fingerprint range of the material extracted from the gas-saturated zone of the blend with CTP 103 can be a sign of the obsolescence of the mesophase properties of extracted material [34].

**Figure 11:**
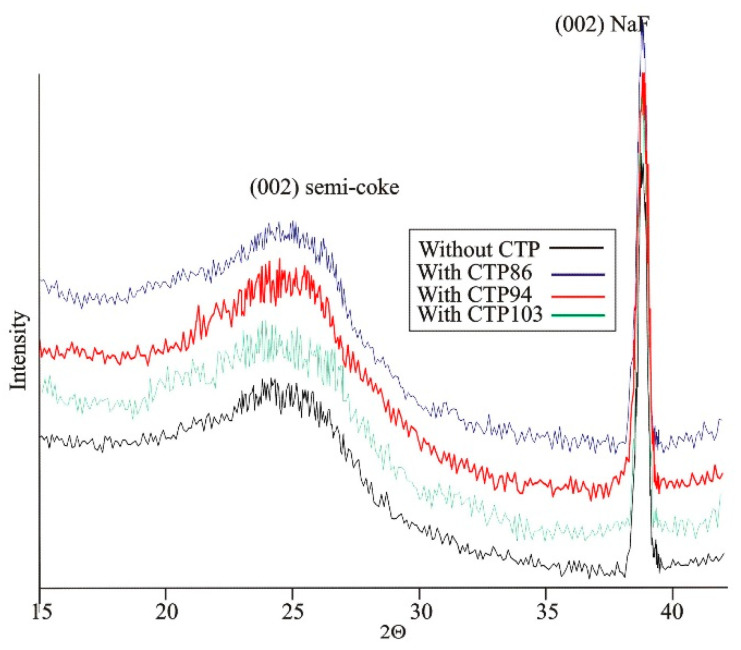
Diffractograms of semi-cokes from carbonized charge of coal and its blends with CTPs.

Taking into account the statement about the role of CTP as a plasticizing agent of the organic coal substance made by Świetlik et al. [7], an XRD investigation of the obtained semicokes was carried out. The degree of ordering of the structure of material in coal blends was evaluated on the basis of an analysis of obtained diffractograms (Figure 11). 

The data in Table 4 imply that all CTPs behave as plasticizing agents with regard to coal and facilitate a better ordering of the structure of semicokes. 

More crystallites (higher value of the *C_cryst_* parameter) are obtained in case of the use of CTP94, but during the formation of cokes with this additive, a greater mass loss will be observed. A better ordering of lamellas inside the crystallites (lower value of parameter *d*_002_) is provided by CTP103 additive; in the semicokes from this blend the average values of interplanar distances are 3.53 Å. In the sequence: coal → blend of coal with CTP86 → blend of coal with CTP94, there is a tendency of increase of the degree of ordering into crystallites. However, the parameters of the structure of semicoke from the blend with CTP103 fall out from that tendency.

The aforementioned suggests that in the blends of coal with CTP86 and CTP94 an increase in the degree of plasticization can be caused by the mesophase properties of the material of own extracts. However, in case of the blend of coal with CTP103, an increase in degree of plasticization can be connected with the activation of the disproportionate processes of hydrogen that are facilitated by CTPs in the role of a hydrogen donor in relation to the organic substance of coal. This suggestion was based on the disappearance of the band corresponding to the presence of bonds of C_ar_-H type in the extracts that can point to a greater contribution of groups with these bonds in the structure of crystallites having a better ordering. 

### 3.4. Physicochemical Investigation of CTPs

**Figure 12:**
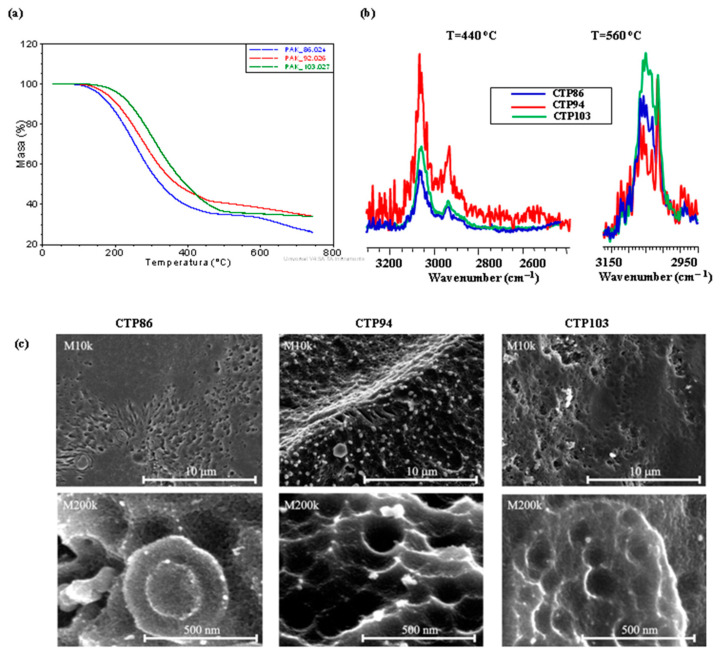
Results of pyrolysis of CTPs: TGA curves of CTPs: (**a**); fragments of normalised FT-IR spectra of volatile products mitted from CTPs at the pyrolysis temperatures of 440 °C and 560 °C (**b**); SEM images of the relief of pyrolyzed CTPs at a temperature of 750 °C (**c**).

Additional investigation was needed in order to explain the causes of the interaction of CTPs with the studied coal as described above. Figure 12 presents the comparison of the curves of changes in the mass loss of CTPs depending on the temperature (Figure 12a), the fragments of FT-IR spectra of CTPs at the temperatures of 440 and 560 °C (Figure 12b), and the SEM images of the relief of the pyrolyzed CTPs (Figure 12c). It follows from the data in Figure 3a that the presence of the same functional groups and the groups of atoms is characteristic of studied CTPs. 

This implied that during pyrolysis CTPs would behave in a similar way but the inclinations in the TGA curves for CTPs having higher softening points would be shifted towards higher temperatures. The mass losses of CTPs are consistent with their softening points to the temperature of approximately 350 °C; more volatile substances are emitted from the pitch having a lower softening point during pyrolysis (Figure 12a). Starting from a temperature of 400 °C, this dependency is disrupted. The height of the FT-IR bands of volatile products normalized with respect to the CO_2_ band shows that at a temperature of 440 °C, CTP94 emits more hydrocarbons than CTP86 and CTP103, and at a temperature of 560 °C, CTP86 emits more volatile hydrocarbons than CTP94. The surface relief of the CTPs pyrolyzed at T = 750 °C differs substantially, and the changes in relief do not indicate any dependence from the softening points of CTPs. The SEM images of the surface relief of CTP86 and CTP103 have more common topological features at magnifications of both M10k and M200k. 

There is an opinion that the softening point and coking yield are connected with the volatility of a CTP [35]. The data presented in Figure 4 and Figure 12a do not allow us to support this opinion. According to another opinion, there should exist some dependency between the amount of released volatile substances in the temperature range of 400–500 °C along with the donor and acceptor abilities of pitch and the development of coke structure. However, the data in Table 3 do not prove this suggestion. It is suggested that the disproportionation processes of hydrogen may be influenced by heteroatoms that are mainly the centres of hydrogen acceptors in the structure of pitch [7].

The results of an EDX microanalysis of the pyrolyzed CTPs presented in Table 5 indicate that an increase in the softening point is accompanied by the rise of the contribution of C atoms in CTPs. The number of other atoms does not point to any dependence from their temperature. In CTP86, a high content of Zn atoms and their dispersion on the surface of pyrolyzed sample draws attention. In the pyrolyzed CTP103 sample, these atoms are observed to a much lesser degree, and in the CTP94 sample it was not identified. 

A great amount of the Fe atoms were determined in some areas of the pyrolyzed CTP103 sample but these atoms were also present in the CTP86 sample. Great amounts of Na atoms were identified in the CTP94 sample but they were also present in pyrolyzed CTP103 sample. 

The analysis of data in Figure 3 and Figure 4c and in Table 3 implies that the pyrolytic behaviour of CTPs can be influenced by the atoms of the metals present in them. This may also influence the interaction of CTPs with coal. It cannot be excluded that the presence of Fe atoms could have contributed to the formation of nano-structures on the surface of the swollen grains in the charge of coal with CTP103 (Figure 6e–h), and that of Zn atoms, contributing to the formation of ‘yarn hanks’ inside the cells of the gas-saturated zone in the blend of coal with CTP86 (Figure 7f). 

## 4. Conclusions

The conducted research does not allow us to state the existence of a direct dependence between the softening point of the pitch and its influence on the thermal behaviour of defrosted coal. Under the influence of CTPs, the thickness of the gas-saturated zone in plastic layer increases in the sequence CTP94 → CTP86 → CTP103. Under the influence of CTP94, the thickness of the plastic layer does not change, CTP86 causes a decrease in the thickness of the plastic layer, and CTP103 causes an increase in the thickness layer.

It was determined that the additions of CTPs to the defrosted coal influenced the yield of volatile products emitted from blends with CTPs to a different degree that is independent from their softening points. CTP, having a softening point of 94 °C, does not change the yield of volatiles, but CTP additives, having a softening points of 86 and 103 °C, cause a decrease in the yield of volatile products of pyrolysis. A greater effect is observed in the case of the CTP86 additive. CTP additives decrease the contribution ratio of saturated and unsaturated hydrocarbons in the composition of volatile products of pyrolysis in the temperature range of 420–500 °C, wherein CTPs, having the softening points of 86 and 103 °C, intensify such decreases. It is suggested that the decrease in the contribution of hydrocarbons in the composition of volatiles of the blend of defrosted coal with CTP86 was caused by their condensation into nano-objects in the cells of plastic mass in the gas-saturated zone. In the blend of coal with CTP103, such decrease in the contribution of hydrocarbons can be connected with the formation of nano-objects on the surface of swollen grains. CTPs influence the composition of extracts from the gas-saturated zone of blends (change the content of compounds with chromophore groups), the topological structure of material of extracts, and their ability to form supramolecular and mesophase structures with organic substance of coal in different ways. This causes the lack of existence of any dependence between the degrees of ordering of obtained semi-cokes and the softening points of CTPs. The ordering changes in the sequence: semi-coke from blend with CTP86→ semi-coke from blend with CTP103→ semi-coke from blend with CTP94.

It is suggested that this lack of dependence between the softening points of CTPs and their influence on the thermal behaviour of coal is caused by the presence of the atoms of metals (Fe and Zn) in pitches that change the course of the carbonization of CTPs themselves and their influence on organic substance of coal in blends with CTPs.

## Figures and Tables

**Table 1:** The characteristics of studied coal.

Proximate Analysis		Ultimate Analysis, %	
W^a^, %	8.5	C^a^	82.2
V^daf^, %	33.0	H^a^	4.82
A^d^, %	7.0	N^a^	1.36
FSI	7.0	S^a^	0.47
RI	77	O^diff^ *	4.15

W^a^—moisture content in analytical sample (air-dry basis); V^daf^—volatiles content in analytical sample (dry, ash-free basis); A^d^—ash content (dry basis); C^a^—carbon atoms content in analytical sample; H^a^—hydrogen atoms content in analytical sample; N^a^—nitrogen atoms content in analytical sample; S^a^—sulphur atoms content in analytical sample; FSI–Free Swelling Index; RI–Roga Index. * calculated by difference O^diff^ = 100—C^a^–H^a^–N^a^–S^a^–A^d^.

**Table 2:** The main characteristics of coal tar pitch.

CTP86			CTP94			CTP103		
T_s_, °C	TI	QI	T_s_, °C	TI	QI	T_s_, °C	TI	QI
86	≥38	≤18	94	≥25	≤9	103	≥18	≤5

T_s_—softening point, TI—toluene insoluble, QI—quinoline insoluble.

**Table 3:** The yield of the material extracted from the zones of plastic layer.

Samples	Condensed Material		Extracted Material	
	350–380 °C	380–410 °C	430–460 °C	470–500 °C
Without CTP [2]	1.35 ± 0.10	2.69 ± 0.03	1.46 ± 0.05	0.83 ± 0.04
With CTP86	1.09 ± 0.06	0.48 ± 0.09	1.35 ± 0.11	1.44 ± 0.07
With CTP94	1.41 ± 0.13	2.79 ± 0.12	2.07 ± 0.09	1.61 ± 0.06
With CTP103	0.69 ± 0.12	1.05 ± 0.11	0.83 ± 0.06	0.98 ± 0.05

**Table 4:** The structural parameters of obtained semi-cokes.

Samples	*d*_002_, Å	*C*_cryst_, %
Semi-coke from charge without CTPs	3.55	50.5
Semi-coke from charge with CTP86	3.55	57.4
Semi-coke from charge with CTP94	3.54	61.3
Semi-coke from charge with CTP103	3.53	59.0

**Table 5:** The elemental composition of pyrolyzed CTPs.

CTP	Elements, wt. %					
	C	O	S	Zn	Na	Fe
CTP86	80.5 ± 2.8	12.3 ± 2.6	1.1 ± 0.5	6.1 ± 4.9	-	0.16 *
CTP94	83.1 ± 1.3	13.5 ± 1.3	0.5 ± 0.2	-	2.87 ± 2.5	-
CTP103	86.2 ± 0.4	9.6 ± 2.5	1.0 ± 0.4	1.1 ± 0.8	1.2 ± 0.8	1.0 ± 0.9

* It occurs only in one of analyzed areas.

## Data Availability

The study did not links to publicly archived datasets analyzed or generated during the study.

## Supplementary Materials

The following supporting information can be downloaded at: https://www.mdpi.com/article/10.3390/ma15249027/s1. Table S1. The values of calculated weight of blends of coal with CTPs.
