# Supplementary material for: The Influence of Coal Tar Pitches on Thermal Behaviour of a High-Volatile Bituminous Polish Coal
Source: Materials (Basel). 2022 Dec 17;15(24):9027. doi: 10.3390/ma15249027 (PMC9785710; doi:10.3390/ma15249027)
Supplement: Supplementary file 1 [file materials-15-09027-s001.zip › materials-2064468-supplementary.pdf]

Table S1. The values of calculated weight of blends of coal with CTPs

Temp. [°C]	Sample weight [%]									
	Coal	CTP86	Coal+CTP86		CTP94	Coal+CTP94		CTP103	Coal+CTP103	
			ex.	calc.		ex.	calc.		ex.	calc.
**100 °C**	98.9	99.6	99.2	98.9	99.8	99.2	98.9	99.9	99.2	98.9
**200 °C**	98.6	86.3	98.7	98.4	91.0	98.8	98.5	96.2	98.9	98.6
**300 °C**	98.3	56.0	98.1	97.5	64.6	98.0	97.6	74.0	98.3	97.8
**350 °C**	97.6	45.5	97.6	96.5	53.3	97.2	96.7	59.9	97.6	96.8
**400 °C**	95.5	39.3	96.5	94.4	46.7	95.3	94.5	48.7	96.1	94.6
**450 °C**	84.3	36.2	90.1	83.4	42.4	86.6	83.5	40.8	88.3	83.5
**500 °C**	76.5	35.1	84.1	75.6	40.8	77.8	75.7	36.6	82.3	75.7
**550 °C**	73.7	34.4	81.2	72.9	39.6	74.7	73.0	35.8	79.5	72.9
**600 °C**	71.8	33.2	78.7	71.0	38.3	72.6	71.1	35.3	77.0	71.0
**650 °C**	70.3	30.7	76.7	69.5	36.9	71.0	69.6	34.9	74.8	69.6
**700 °C**	69.2	28.1	75.0	68.3	35.4	69.8	68.5	34.5	73.0	68.5
